# The influence of tourism and hospitality students’ perceived effectiveness of outcome-based education on their VUCA skills

**DOI:** 10.1038/s41598-023-35186-5

**Published:** 2023-05-18

**Authors:** Yanbing Guo, Qiaoyan Zhao, Zhe Cao, Shaosheng Huang

**Affiliations:** 1 School of Culture Tourism and Journalism Arts, Shanxi University of Finance and Economics, No. 140, Wucheng Road, Taiyuan, 030006 Shanxi Province People’s Republic of China; 2 Faculty of Business Administration, Shanxi University of Finance and Economics, Taiyuan, 030006 People’s Republic of China

**Keywords:** Psychology, Human behaviour

## Abstract

The mismatch between supply and demand in the tourism and hospitality labor markets becomes increasingly serious in the past few years. The main reason for such a problem is that tourism and hospitality students are equipped with academic knowledge but lack VUCA skills. VUCA are the acronyms of volatility, uncertainty, complexity, and ambiguity. However, little research has explored the antecedent mechanism of tourism and hospitality students’ VUCA skills. Hence, the purpose of the present study is to clarify the leading factors that would enhance tourism and hospitality students’ perceived VUCA skills. This study collected data by distributing questionnaires to senior students majoring in tourism and hospitality management (THM) from five universities in China. The results show: First, students’ perceived the effectiveness of outcome-based education (PEOBE) has a significant effect on their perceived VUCA skills and self-concept including cognitive self-concept (CSC) and affective self-concept (ASC). Second, THM students’ CSC is positively related to their perceived VUCA skills. Finally, the relevance of ASC and students’ perceived VUCA skills was not significant. The present study further justified that PEOBE is the prepositive variable of THM students’ cognitive self-concept, supporting the linkage effect of “PEOBE → CSC → self-efficiency → perceived VUCA skills”. From the practical implications, this study focuses on OBE as the entry point to explore the antecedent mechanism of THM students’ perceived VUCA skills, supplying a basic formulation of educational reform policies for the administrative department of higher education around the world.

## Introduction

With the rapid growth of the tourism economy, the demand for a workforce in the tourism industry becomes more urgent. Many universities and colleges in China established the majors like tourism and hospitality management (THM), providing an increasing number of graduates year by year. However, less than 30% of THM students choose to work in the tourism and hospitality industry after graduation according to the “China Labor Statistics Yearbook”, indicating that there has been an enormous gap between supply and demand in the labor market of the TH industry. Most THM students engaged in other fields (e.g., finance, accounting & sales) after graduation rather than being tour guides, operators, tourism planners, etc. Even in the postgraduate entrance examination, most THM students gave up THM majors. Surprisingly, these phenomena have been existing for nearly two decades. Newly employed THM graduates seem to have academic equipment, but they are not capable of coping with various intractable problems in their future careers. With the advent of the VUCA era (i.e., volatility, uncertainty, complexity, and ambiguity)^1–3^, tourism enterprises have been facing various unprecedented challenges. Therefore, staff members with VUCA skills are increasingly favored by the tourism industry. Just under this background, a growing number of educators appeal for the reverse design and customization of students’ curricula according to their future career requirements. Thus, the OBE (i.e., outcome-based education) theory proposed by Spady^4^ has again attracted the attention of the current academic circles. Indeed, OBE has been widely advocated and implemented by some universities to improve their students’ VUCA skills. The specific forms of OBE implemented by universities include educational travel, experiential education, internships, extracurricular activities, etc., which are methods for planning, delivering, and evaluating instructions, which require administrators, tutors, and students to focus their attention and efforts on the desired results of education^5^.

In the engineering education, Syeed et al.^6^ believed that the OBE is an approach to education in which decisions about the curriculum are driven by the exit learning outcomes that the students should perform in their professional life. Zamir et al.^7^ pointed out that the overall transformation from non-OBE to OBE has proved the new educational system superlative in student learning, subject delivery, and teaching technique. In the nursing education, Tan et al.^8^ investigated the influence of OBE on students’ knowledge acquirement, skills performance, attitudes, and behaviors with the ethnography method. In the rencent year, the OBE framework was gradually introduced into THM education. Since the objective of THM education is to enable students to equip with VUCA skills, exploring the formation mechanism of students’ VUCA skills has become an important scientific issue. The present study selects five colleges that have been implementing OBE for several years to explore the influence of students’ perceived effectiveness of OBE on their perceived VUCA skills. Notably, extracurricular activities are the specific form of OBE as noted, and students’ leadership covers the abilities to analyze their own merits, confidence, responsibility, communication, decision-making, problem-solving, and career plan^9^, which are the important sectors of students’ VUCA skill set. Students learning experiences affect self-efficacy and result in expectations, which, in turn, influence their interests, personal goals, career choice, and performance^10^. Therefore, this study holds that OBE has certain correlations with students’ VUCA skills. A recent study argued that self-efficacy is the prepositive variable of students’ VUCA skills^11^, and self-concept, whereas, is widely regarded as the antecedent of self-efficacy. Thus, the present study intends to clarify the relationship between OBE and students’ perceived VUCA skills by incorporating self-concept as the mediating variable into the research model. Specifically, the objectives of the present study are as follows:

1. To explore the influence of THM students’ PEOBE on their perceived VUCA skills;
2. To investigate the effect of THM students’ PEOBE on their self-concept;
3. To confirm the influence of THM students’ self-concept on their perceived VUCA skills;
4. To examine the mediating role of self-concept in the path of THM students’ PEOBE on their perceived VUCA skills.

The contributions of this research are as follows: From the perspective of the method, the current study utilized SEM (Structural Equation Modeling) to justify the fruits that the ethnography method achieved before, namely that students’ perceived effectiveness of outcome-based education (PEOBE) influences their skills, performance, and knowledge acquirement. From the perspective of theoretical contributions, the present study justified that THM students’ perceived effectiveness of OBE could not only directly lead to their VUCA skills, but also significantly promotes their VUCA skills through the full mediating effect of CSC rather than ASC, which indicated that THM students’ perceived effectiveness of OBE is the prepositive variable of self-concept, supporting the linkage effect of “PEOBE → self-concept → self-efficiency → students’ perceived VUCA skills”. Third, from the perspective of practical implications, this study focuses on OBE as the entry point to explore the antecedent mechanism of THM students’ perceived VUCA skills, supplying a basic formulation of educational reform policies for the administrative department of higher education around the world. Finally, a growing number of Chinese universities canceled THM majors in succession, since students majoring in THM rarely engage in the work relating to their previous major after graduation. Thus, studying this issue can be enlightening for curriculum improvements and designs for colleges or universities in China.

## Literature review

### OBE

OBE is a method of curriculum setting, teaching implementation, and talent training according to the preset educational objectives and learning achievements^4^. Killen (2000) pointed out that OBE emphasizes what learners should know, understand, and show and how to adapt to future life roles^12^. OBE has been widely implemented and advocated in various fields, such as medical science, nursing, pharmacy, and engineering^13–16^ in the US, the UK, New Zealand, Malaysia, Australia, South Africa, and the Philippines^17^. In the past few years, OBE has been extended to leisure, sport, and THM education. For instance, Arcodia and Dickson^18^ pointed out that experiential education has a strong potential to be beneficial to students. In addition, the educational tour is a particularly useful learning approach in the field of THM education. Felicen^19^ evaluated the effectiveness of OBE in THM education from curriculum relevance, curriculum organization, and teaching activities. As indicated, either experiential education or an educational tour is the characterization of OBE.

There is compelling evidence showing that the OBE framework also faced various challenges and queries although a number of proponents regarded it as a successful and meaningful form of a learning experience. For instance, Berlach and O'Neill^20^ indicated the little agreed understanding regarding the core of the curriculum has resulted in the absence of a reference point for developing satisfactory examination protocols under an OBE framework. Shaheen^21^ systematically analyzed twelve challenges that the OBE framework faces in Pakistan, such as acceptance challenge, maintaining the quality of education, and paradigm shift.

While some scholars believed that the OBE framework is defective, concentrating on the promoting function of OBE in training students’ abilities and quality development has been a concern for educators for a long time. In recent years, various universities and colleges in China have begun to implement OBE to cultivate THM students, aiming to enhance their VUCA skills and narrow the gap between supply and demand in the labor markets. In June 2013, China was admitted as a signatory member of the “Washington Agreement,” which marked the beginning of OBE implementation in China.

### VUCA theory

The notion of VUCA was introduced by the US Army War College to portray the volatile, uncertain, complex, and ambiguous world, which derived from the end of the Cold War^22^. Subsequently, strategic business leaders adopted it to describe a chaotic, volatile, and rapidly changing business environment^23^. Recently, VUCA has gradually moved from business to the education field. For instance, educational visions (e.g., OECD 2030) hope that future students will develop skills and attitudes that will enable them to succeed in a VUCA world^24^. Additionally, policy documents from UNESCO and OECD have been emphasizing the demand to prepare students for what has been termed a VUCA world^25^. The tourism industry is vulnerable and more likely to be struck by wars, epidemics, policies, and disasters. All of those factors may make THM students face VUCA problems in their future careers. Hence, educational administrative departments and institutions of higher education have to equip THM students with VUCA skills to better tackle VUCA problems in their future careers. With regard to the definition of VUCA skills, Intagliata and Small^26^ indicated that VUCA skills include self-awareness, knowledge of the business, innovative and critical-thinking skills, collaboration, etc. Horstmeyer (2019) suggested that VUCA skills involve curiosity, creativity, and disruption tolerance^27^.

Overall, previous studies have mainly discussed the definition and elements of leaders' VUCA skills. Few researchers have concentrated on students’ VUCA skills, especially THM students. The present study holds that THM students might face a chaotic, turbulent, and rapidly changing business environment. Therefore, it is necessary and urgent to explore the antecedent mechanism and components of THM students’ VUCA skills.

### Self-concept

Self-concept refers to the general concept that individuals hold about themselves in different aspects like behavior, competence emotion, and attributes, and these aspects are formed by personal experience and interpretation of the external environment^28^. Turner et al.^29^ suggested that self-concept is a cognitive component of the psychological system or process involving the self, and it is a series of cognitive representations of individual to self. Self-concept is an important theory of cognitive psychology, which has been introduced to fields such as marketing, advertising, tourist behaviors, and education.

In educational contexts, self-concept could be divided into cognitive self-concept (CSC) and affective self-concept (ASC)^30^. The CSC is pertaining to one’s sense of competence^30,31^. The ASC, whereas, is more related to individuals’ interests^31^. A key question for THM education is how the dual constructs of self-concept work in students’ knowledge and skills, especially for VUCA skills.

### Hypothesis development

#### PEOBE and perceived VUCA skills

Participating in extracurricular activities could cultivate students’ interpersonal communication and social adapt ability^32^. Therefore, higher education began to shift from “process” as the center to “outcome” as the guidance. Specifically, universities have reversely designed their curriculum contents and teaching methods based on a comprehensive understanding of the demands of the labor markets, namely that starting with a blueprint of what is necessary for students to do and then planning the courses, teaching, and evaluation to guarantee that the learning thoroughly takes place^33^. The OBE leads to the development of nursing students’ competencies more than the traditional method^34^. Tan et al. (2018) further justified the effectiveness of OBE in promoting nursing students’ abilities, showing that OBE methods are positively related to nursing students’ knowledge and skills^6^. OBE programs can not only empower students in their academic fields but also narrow the gap between universities and the career world^5^. Yildiz^35^ believed that outdoor activities would enhance participants' self-consciousness and socialization experiences. As experiential education, outdoor activities enable students to participate, improve learning efficiency and develop the professional abilities expected by enterprises^36–38^.

Overall, OBE is closely related to the development of students’ comprehensive skills (i.e., abilities to analyze their own merits, confidence, responsibility, communication, decision-making, problem-solving, and career plan). It can provide students with experience and situations and create basic practical opportunities that are important prerequisites for improving leadership^39,40^. Hence, this study proposes the following hypothesis based on the above discussion:

##### H1

PEOBE has a significant effect on THM students’ perceived VUCA skills.

#### PEOBE and self-concept

Ample evidence shows that a student's external environment is the most important frame of reference for the development of self-concept^41^. Higher education implemented OBE through various approaches, such as educational tours, experiential education, extracurricular activities, and internship, which provide students with an external environment for creating their self-concepts. Bloomfield (2011) ever pointed out that students’ participation in extracurricular activities could improve their general self-concept^42^, that is, students who participate in any kind of extracurricular activities have a higher level of general self-concept than those who do not. Thus, the positive development experience provided by extracurricular activities can significantly predict students’ general self-concept.

As noted, self-concept can be divided into two sectors, namely, CSC and ASC. Most of the acquirements from OBE might lead to the improvement of students’ CSC, that is, students are inclined to believe that they have held the abilities to study and work. Moreover, implementing OBE is likely to contribute to student’s academic interest, which is the core connotation of students’ ASC according to the above discussion^31^. Arcodia et al.^43^ proposed that THM education should begin with field trips and provoke students’ motives and studies through tours, entertainment, and novelty. Research shows that experiential education is positively related to student's academic satisfaction^44,45^, which might directly lead to the improvement of their academic interests, self-efficacy, and self-concepts. Accordingly, the present study proposes the hypotheses:

##### H2

PEOBE has a significant effect on CSC.

##### H3

PEOBE has a significant effect on ASC.

#### Self-concept and perceived VUCA skills

Stiehm (2003) believed that the modern combat military environment comprises VUCA situations^46^. Therefore, military leaders need a stable self-concept to adjust their thoughts, structure, and use of technology to face certain situations; and they should be adaptable and innovative, able to make adjustments based on continuous evaluation^47^. On this basis, individuals’ self-concepts have been given great significance in developing their VUCA skills.

Self-concept accelerates the expected outcomes of various aspects^48^. Specifically, Positive self-concepts help students succeed in assignments, social situations, and daily lives, which shows that students’ confidence is essential for a series of ideal outcomes^49^. The positive correlation between self-concepts and career intentions, education aspirations, and resilience has been acknowledged by many researchers^50–52^. Choy and Yeung^49^ discussed the effects of CSC and ASC, arguing that the former is positively related to THM students’ operative skills, resilience, and competence, whereas the latter has a significant effect on their career choice and aspiration. Some scholars believed that CSC is more about academic and performance results^53–55^, while ASC has more relationship with motivation and engagement^31^. Jansen et al.^56^ proposed that students having a strong ASC are more likely to dedicate attention and time to specific issues of interest and might realize a higher accomplishment. According to social cognitive theories, subjective beliefs such as self-concept and self-efficacy play an important role in individuals’ growth and development^57^. Boe et al.^47^ claimed that self-concept is the prepositive variable of self-efficacy, and self-efficacy has a significant effect on THM students’ VUCA skills. Given all these existing studies, the present research proposes the hypotheses:

##### H4

CSC has a significant effect on THM students’ perceived VUCA skills.

##### H5

ASC has a significant effect on THM students’ perceived VUCA skills.

**Figure 1:**
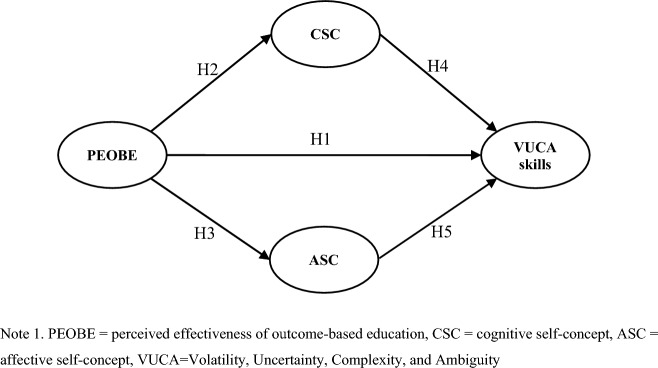
Research model.

Figure 1 shows the conceptual model.

## Methodology

### Measurement

Six questions were set to gauge the PEOBE (e.g., “The course helped me to develop relevant subject knowledge” and “The course was implemented according to the approved curriculum”)^19^. The measurement of self-concept was divided into two parts: CSC and ASC. The CSC was appraised with three questions (e.g., “I am competent in all technical skills within my area of duty”), and the ASC was gauged by three questions as well (e.g., “I am interested in my preferred tourism and hospitality discipline”)^49^. Referring to the research of Kautish et al.^11^, the present study utilized a self-evaluation scale to assess THM students’ perceived VUCA skills, which were measured with 10 items (e.g., “I exploit my inner drive to build and grow things” and “I have the ability to make sense of clutter”). A Likert scale of five points was applied to gauge the variables without reverse coding items: 1 equals “strongly disagree”, and 5 equals “strongly agree”.

### Data collection

Senior students (i.e., last year of bachelor's education) of THM from five universities in China were chosen by our researchers as the survey samples. The reason why we chose them as samples are as follows. First, all five universities implemented OBE, inversely designing curricula according to the labor demand of the tourism industry to improve THM students’ comprehensive skills to adapt them to VUCA environments. Second, the students majoring in THM in these universities were informed that they would experience OBE when they entered the campus. Their curricula were inversely designed by the educators according to their career planning scale, namely that these students understand the OBE framework and are aware that they are educated under the OBE. Finally, these senior students had experienced four years of OBE in colleges and had finished their internship and educational trip. Therefore, they were able to perceive the effectiveness of OBE in their universities and whether they already had the VUCA skills. Our researchers first contacted the tutors in charge of the faculty of tourism and hospitality management at the above universities by E-mail. Then, they were told the topic, purpose, significance, and requirements of our research. After asking for their consent, the questionnaires were distributed to the graduating class students, namely the students in the last year of their bachelor's education. Before filling out the questionnaire, the students were asked whether they had experienced OBE like educational travel, experiential learning, or internship. Those who ticked ‘No’ were not chosen by the researchers. To reduce the sampling error, students suspending their studies or delaying their graduation would be not included in the samples. Universities in China were encouraged to conduct online teaching due to the COVID-19 pandemic. Thus, some of the questionnaires were distributed by a network platform (i.e., Questionnaire Star), and others were handed out by our researchers at sites. Our group spent 20 days collecting data from March 20 to April 9, 2022. 450 questionnaires were handed out by our researchers, and 386 of them were qualified and valid. The proportion of valid questionnaires accounted for 85.7%.

### Data analysis

First, we described the demographic profiles. Then, a one-way ANOVA method was utilized to test the difference in PEOBE, CSC, ASC, and VUCA skills among individuals. Third, we utilized Cronbach's α values to evaluate the reliability of internal consistency. Apart from that, a CFA model was constructed to assess reliability and validity. Finally, an SEM was constructed to examine the proposed hypotheses.

### Demographic profile

Of the total respondents, 188 were males and 198 were females, constituting 48.7% and 51.3%, respectively. With regard to their majors, 195 were majoring in hospitality management and 191 were majoring in tourism management, constituting50.5%, and 49.5%, respectively. 25.4% of the total respondents came from University E, followed by University A (23.3%), University B (19.6%), and University C (19.2%). The proportion of respondents from University D was the least, accounting for 12.5%. From the perspective of internship enterprises, 109 students had their internships in scenic areas, reaching 28.2%. There was little difference in the proportion of students whose internships were in transportation departments and Resteurants, reaching 25.4% and 23.8%, respectively. The proportion of students who had internships in travel agencies was the same as that of other divisions, at 5.2% (Table 1).

**Table 1:** Demographic profile of the respondents.

	N = 386	%
Gender		
Male	188	48.7
Female	198	51.3
Major		
Hospitality management	195	50.5
Tourism management	191	49.5
Universities		
University A	89	23.3
University B	75	19.6
University C	74	19.2
University D	48	12.5
University E	100	25.4
Types of internship enterprises		
Travel agencies	20	5.2
Star hotels	47	12.2
Resteurants	92	23.8
Scenic areas	109	28.2
Transportation	98	25.4
Others	20	5.2

### One-way ANOVA test

Considering that students with different demographic profiles may have significant differences in PEOBE, CSC, ASC, and VUCA skills, this study selected four factors, including gender, major, university, and internship enterprises, and used one-way ANOVA to test the perception differences of PEOBE, CSC, ASC and VUCA skills among different groups. As shown in Table 2, all the *p* values are much higher than 0.05, indicating that there is no significant difference in the impact of demographic profiles on PEOBE, CSC, ASC, and VUCA skills. Hence, it is reasonable to believe that the OBE framework has been implemented at the same level in all these five universities (Table 2).

**Table 2:** One-way ANOVA test of demographic profiles.

Demographic profiles	Types	PEOBE	CSC	ASC	VUCA skills
Gender	Male (48.1%)	3.18	3.67	3.34	3.37
	Female (51.3%)	3.25	3.66	3.40	3.57
	*p*	0.38	0.40	0.55	0.39
Major	Hospitality Management (50.5%)	3.19	3.59	3.31	3.47
	Tourism Management (49.5%)	3.25	3.74	3.43	3.48
	*p*	0.49	0.12	0.20	0.86
Universities	University A (23.3%)	3.45	3.73	3.33	3.45
	University B (19.6%)	3.43	3.68	3.45	3.39
	University C (19.2%)	3.38	3.52	3.41	3.48
	University D (12.5%)	3.55	3.58	3.58	3.44
	University E (25.4%)	3.39	3.66	3.51	3.66
	*p*	0.39	0.54	0.23	0.55
Types of internship enterprises	Travel agencies	3.33	3.50	3.13	3.08
	Star hotels	3.07	3.76	3.48	3.54
	Resteurants	3.20	3.59	3.34	3.47
	Scenic areas	3.22	3.69	3.36	3.40
	Transportation	3.24	3.71	3.36	3.55
	Others	3.40	3.58	3.60	3.79
	*p*	0.73	0.78	0.65	0.10

### Ethical approval

We confirm that all methods were carried out in accordance with relevant guidelines and regulations. We confirm that all experimental protocols were approved by Shanxi University of Finance and Economics ethics committees/IRB.

### Informed consent

We confirm that informed consent was obtained from all subjects and/or their legal guardian(s).

## Results

### Evaluation of the CFA model

The results show that the CFA model is in good agreement with the data (*χ*^2^ = 306.321, *df* = 203, *χ*^2^/*df* = 1.509, RMSEA = 0.036, CFI = 0.981, IFI = 0.981, TLI = 0.978). Tables 3 and 4 show the reliability and validity of the model with CFA model. Internal consistency reliability is determined with Cronbach's α of each latent variable, distributing between 0.80 and 0.94, higher than the suggested critical value of 0.7. Hence, all the latent variables are reliable and credible. The convergent validity is determined by C.R., average variance extracted (AVE), and factor loadings. First, all factor loadings distribute between 0.72 and 0.84, which are much greater than the recommended critical value of 0.6, significant at a 1% level. Second, the C.R. of each construct is much higher than the recommended critical value of 0.7 (0.80–0.94)^58^. Third, the AVE of each construct is higher than the suggested cut-off point of 0.5, ranging from 0.58 to 0.65 (≥ 0.5). Therefore, the convergent validity of the measurement tool is qualified. In addition, all the AVE values are much greater than the square of the correlation coefficient between each latent variable, indicating that the scale has qualified discriminant validity^59^.

**Table 3:** Measurements, factor loading, C.R., and AVE.

Measures	Loadings	Cronbach's α	C.R	AVE
Perceived effectiveness of outcome-based education (PEOBE)		0.91	0.91	0.65
The educational travel helped me to develop relevant subject knowledge	0.84			
The internship was implemented according to the approved curriculum	0.82			
Teaching & Learning Activities (TLAs) such as practical, educational tour, etc. were useful and relevant	0.80			
Assessment methods to be used were told at the beginning of the course	0.83			
Available facilities in the classrooms were satisfactory	0.75			
The teachers were available for consultation whenever needed	0.78			
Cognitive self-concept (CSC)		0.83	0.83	0.62
I am competent in all technical skills within my area of duty	0.82			
I am doing well in my major	0.73			
I am capable of doing good in my current discipline	0.80			
Affective self-concept (ASC)		0.80	0.80	0.58
I am interested in my preferred hospitality and tourism discipline	0.74			
I like studying in my major	0.82			
I enjoy majoring in hospitality and tourism management	0.72			
VUCA skills		0.94	0.94	0.64
I exploit my inner drive to build and grow things	0.80			
I have the ability to make sense of clutter	0.79			
I am able to turn dilemmas into advantages	0.79			
I learn from unfamiliar environments by immersing myself in the process	0.80			
I try to see and learn things from nature’s point of view	0.85			
I am calm under tense situations & facilitate constructive engagement	0.81			
I am open and authentic	0.80			
I have the ability to quickly create early versions of practical innovations	0.79			
I use current media to create & engage change networks	0.80			
I endorse shared assets that benefit others & heighten competition (carpooling, sharing culture etc.)	0.76			

*C.R.* composite reliability, *AVE* average variance extracted.

Goodness-of-fit statistics for the measurement model**:** *χ*^*2*^ = 306.321, *df* = 203, *χ*^*2*^*/df* = 1.509, RMSEA = 0.036, CFI = 0.981, IFI = 0.981, TLI = 0.978.

All standardized factor loadings were significant (*p* < 0.001).

**Table 4:** Measurement model correlations.

	PEOBE	CSC	ASC	VUCA skills
PEOBE	1			
CSC	0.22^a^ (0.05^b^)	1		
ASC	0.16 (0.02)	0.11 (0.01)	1	
VUCA skills	0.22 (0.05)	0.22 (0.05)	0.2 (0.04)	1
AVE	0.65	0.62	0.58	0.64
Mean	3.38	3.73	3.53	3.45
S.D	0.81	0.86	0.84	0.69

*PEOBE* perceived effectiveness of outcome-based education, *CSC* cognitive self-concept, *ASC* affective self-concept.

Goodness-of-fit statistics for the measurement model: *χ*^*2*^ = 306.321, *df* = 203, *χ*^*2*^*/df* = 1.509, RMSEA = 0.036, CFI = 0.981, IFI = 0.981, TLI = 0.978.

^a^Correlations between variables.

^b^Squared correlations between variables.

### Evaluation of the SEM

**Figure 2:**
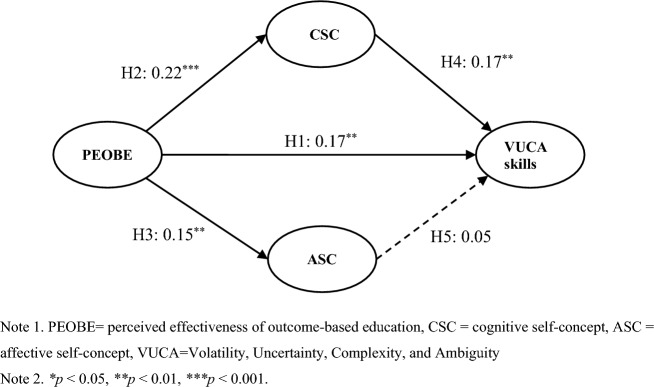
Results of the structural equation modeling.

Table 5 and Fig. 2 show the results of the SEM. First, the results show that the model-fit coefficient supports the SEM (*χ*^2^ = 314.043, df = 204, *χ*^2^/*df* = 1.539, RMSEA = 0.037, CFI = 0.979, IFI = 0.979, TLI = 0.977). Second, all other hypotheses, H1–H4, are supported, except H5. As shown in Table 4 and Fig. 2, PEOBE has a significant effect on VUCA skills (*β*_PEOBE→VUCA Skills_ = 0.17, *p* < 0.01, *t* = 3.08). Thus, H1 is supported. With reference to the relationship between PEOBE and self-concept, the data indicate that PEOBE has a positive effect on CSC and ASC, respectively (*β*_PEOBE→CSC_ = 0.22, *p* < 0.001, *t* = 3.81;* β*_PEOBE→ASC_ = 0.15, *p* < 0.01, *t* = 2.68). Thus, H2 and H3 are established. With regard to the relevance of self-concept and VUCA skills, the data indicate that CSC has a significant influence on VUCA skills (*β*_CSC→VUCA Skills_ = 0.17, *p* < 0.01, *t* = 2.94), but the correlation between ASC and VUCA skills is insignificant (*β*_ASC→VUCA Skills_ = 0.05, *p* > 0.05, *t* = 0.87). Hence, H4 is supported, whereas H5 is rejected.

**Table 5:** Hypotheses testing.

Hypotheses	Paths	Path coefficient	*t*-Value	Supported
H1	PEOBE → VUCA skills	0.17	3.08**	Yes
H2	PEOBE → CSC	0.22	3.81***	Yes
H3	PEOBE → ASC	0.15	2.68**	Yes
H4	CSC → VUCA skills	0.17	2.94**	Yes
H5	ASC → VUCA skills	0.05	0.87	No

Mediating path	Path coefficient	Confidence interval (95%)		Significance
		Lower	Upper	
PEOBE → CSC → VUCA skills	0.03	0.01	0.08	**
PEOBE → ASC → VUCA skills	0.02	−0.01	0.03	0.27 (*p *> 0.05)

Goodness-of-fit statistics for the measurement model: *χ*^*2*^ = 314.043, *df* = 204, *χ*^*2*^*/df* = 1.539, RMSEA = 0.037, CFI = 0.979, IFI = 0.979, TLI = 0.977.

*p < 0.05, **p < 0.01, ***p < 0.001.

Table 5 shows the mediating analysis. The results show that CSC plays a partial mediating effect between PEOBE and students’ VUCA skills. The mediating effect reaches 0.03, and the confidence interval of 95% is between 0.01 and 0.08, excluding 0. Hence, the mediating effect is significant, manifesting that the partial mediating effect is significantly supported.

## Discussion

Whether educational units should implement the OBE framework has caused a heated debate in academia. Some scholars believed it has built a bridge between the “future” and the “present” for students. However, numerous researchers have even made major criticism of the OBE framework. The reason might be that scholars conducted their research based on various objects, circumstances, and contexts, leading to the divergence. This study takes senior THM students from five universities in China as cases to prove the applicability of the OBE framework in domestic THM education. Overall, the present study has explored the influence of THM students’ PEOBE on their perceived VUCA skills by taking CSC and ASC as mediating roles. All other hypotheses (e.g., H1–H4) were supported, except H5.

First, this study found that THM students’ PEOBE has a significant effect on their perceived VUCA skills. This conclusion highly matches the previous research that argued the positive effects of OBE on nursing students’ competencies and skills^8,34^. In comparison with these articles, this study focused on examining the leading factors in the improvement of THM students’ VUCA skills. THM students might have plenty of academic knowledge upon graduation but lack the application of knowledge into practice, especially in problem-solving, leadership, communication, and critical thinking skills, which are precisely the skill set required by the labor market. Hence, clarifying the relationship between PEOBE and perceived VUCA skills has great significance to the curriculum design among various universities.

Second, THM students’ PEOBE is positively related to THM students’ CSC and ASC. This result further confirms the positive effect of OBE implementation on students’ construction of self-concept. Educational travel, internship, and extracurricular activities have been widely advocated and implemented by various universities to put the OBE philosophy into practice. Li and Wang^9^ mainly suggested that extracurricular activities play an important role in constructing students’ general self-concepts. However, extracurricular activities are supposed to have a broader extension, including various experiential activities, such as educational travel and internship. This study extended extracurricular activities to other types, such as educational travel and internship, and explored the effect of THM students’ perceived effectiveness of educational travel and internship on their self-concepts. In addition, Li and Wang^9^ did not discuss this issue by dividing self-concept into two dimensions (CSC and ASC). However, this study divided general self-concept into CSC and ASC and confirmed that PEOBE is positively related to each of them, thereby providing theoretical implications to self-concept theory.

Third, CSC has a significant influence on THM students’ VUCA skills, but the relationship between ASC and THM students’ VUCA skills has not been justified. As noted, CSC refers to individuals’ senses of competence, whereas ASC refers to individuals’ senses of interests^30,31^. Notably, Choy and Yeung^49^ pointed out that CSC is more pertaining to students’ performance-based and competence-based skills (e.g., operative skills and resilience). They also stated that ASC is more pertaining to non-performance-based skills (e.g., career choice). However, as indicated, VUCA skills include self-awareness, knowledge of the business, innovative and critical-thinking skills, collaboration, curiosity, creativity, and disruption tolerance^26,27^, which are a set of abilities belonging to performance-based and competency-based outcomes. Therefore, the relationship between CSC and VUCA skills has been justified, while the relevance of ASC and VUCA skills is not significant.

Finally, CSC plays a partial mediating role in the relationship between PEOBE and VUCA skills. That is, the stronger the effectiveness of OBE perceived by students is, the more they will think they have competence and self-confidence, and the more they will feel that they have had VUCA skills. Kautish et al.^11^ revealed that students’ career self-efficacy has a significant effect on their VUCA skills. Additionally, evidence shows that self-concept is the prepositive variable of self-efficacy^47^. The present study further examined the pre-leading factor that can improve students’ self-concepts (i.e., PEOBE). The result shows that PEOBE is an important role in constructing -M students’ CSC, supporting the linkage effect of “PEOBE → self-concept → self-efficiency → VUCA skills”.

### Conclusion

The mismatch between supply and demand in the labor market has caused a heated debate in academia. To address this issue, a large number of universities and colleges have begun to inversely design their courses and education methods to cultivate their students to meet the demand of the labor market. OBE implies that the product defines the process, that is, educational institutions ought to focus more on the outcomes instead of the process. Hence, the traditional curriculum has been outdated in the VUCA era. Students should not only hold academic knowledge but also master VUCA skills to address VUCA problems in their future careers. Thus, experiential teaching and learning (e.g., internship, educational travel, and extracurricular activities) have been widely advocated and implemented by various universities in China to develop THM students’ VUCA skills. Currently, few studies have explored the formation mechanism of THM students’ VUCA skills. Thus, the present investigation examined the THM students’ PEOBE on their VUCA skills by incorporating CSC and ASC as mediating roles, sampling the THM students from five universities in China. The results show that PEOBE is highly related to THM students’ VUCA skills, CSC, and ASC. Moreover, CSC is positively related to VUCA skills, whereas the correlation between ASC and VUCA skills is insignificant. Finally, CSC is an important mediating variable in the path of PEOBE influencing VUCA skills. This study not only focused on the relationship between PEOBE and VUCA skills but also enlightened several theoretical and practical implications for educational and academic circles.

### Theoretical implications

Two theoretical implications could be deduced from the present research. First, the present study stretched the chain antecedents of students’ VUCA skills. Kautish et al.^11^ claimed that self-efficacy has a significant effect on students’ VUCA skills or has a significant effect through the mediating role of social support. Moreover, self-concept has been regarded as a prepositive variable of self-efficacy^47^. This study further confirmed that THM students’ PEOBE is highly related to their CSC. Thus, THM students’ VUCA skills could be derived from PEOBE through the chain mediating effects of self-concept, self-efficacy, and social support. Second, this is an empirical study of OBE philosophy affecting VUCA skills from the perspective of THM students’ CSC and ASC. Tan et al.^8^ utilized observation and interviews to investigate the influence of OBE affecting students’ knowledge acquirement, skills performance, attitudes, and behaviors. Felicen^19^ used a descriptive design to assess the effectiveness of OBE implementation. However, the present study utilized SEM to explain the internal mechanism of THM students’ PEOBE affecting their VUCA skills.

### Managerial implications

There are four managerial implications drawn from the present study. First, the administrative department of higher education should actively promote the educational reform of tourism and hospitality majors, implement outcome-based education policy, and compose an OBE syllabus based on curriculum overview and objectives, core competence weight, teaching methods, teaching evaluation, schedule, reference books, etc. Second, educators are supposed to track tourism and hospitality students’ career plans at different stages, understand their expectations for future careers through interviews and questionnaires, customize students’ curriculum content according to their career planning, and cultivate talents with a targeted view. When students’ career planning changes, educators should actively adjust the curriculum to meet the development needs of tourism and hospitality students. For instance, educators should use career planning scales on students in each new semester to understand THM students’ career plans or interests, and reversely design their courses, grouping students with common career plans together for teaching. Third, universities and colleges should establish affiliated hotels, travel agencies, tourism planning companies, etc., if conditions permit, to provide convenience for students’ outcome-based education. Each department should be equipped with professional technical training teachers to provide skill training for students, helping them acquire direct experience. Finally, THM students ought to actively cooperate with college educators to implement OBE frameworks like educational travel, experiential learning, and internship to enhance the perceived effectiveness of OBE. Improving students’ perceived effectiveness of OBE could provoke their cognitive self-concepts and improve their VUCA skills. Therefore, THM students need to submit their career plans and interests to educators in the form of written reports, and provide factual feedback to facilitate educators to tailor the courses according to students’ demands.

### Limitations and future research

The current research has several limitations. First, this study utilized a self-evaluation scale to assess THM students’ VUCA skills instead of the other-evaluation method. Therefore, the conclusions drawn from the current study might be different from those with other-evaluation scales. Therefore, it is necessary to invite experts or industry practitioners to evaluate THM students’ VUCA skills in future research and compare the results with this study. Second, this study explored the direct effect of PEOBE affecting perceived VUCA skills and the mediating roles of CSC and ASC. Students’ self-concepts and skills might be affected by the participants' backgrounds. According to SCCT, personal characteristics (i.e., personality, gender, nation, abilities, and health status) and previous background factors (i.e., social, economic, and cultural status) could influence the learning experience, self-efficacy, outcome expectation, interests, and performance. Therefore, in the future, we should take personal characteristics and previous background factors as moderating variables to examine the effect of PEOBE on perceived VUCA skills. Finally, this study utilized a comprehensive scale to measure THM students’ PEOBE rather than a subscale. Some of the previous literature has used subscales, including the relevance of the course, course organization and ILOs, teachers and TLAs, assessment, learning environment, and counseling to measure PEOBE. Hence, future research could explore the effects of various dimensions of PEOBE affecting CSC, ASC, and perceived VUCA skills.

## Data Availability

The datasets used and analyzed during the current study are available from the corresponding author upon reasonable request.

## References

[1] Sarkar A (2016). We live in a VUCA World: The importance of responsible leadership. Dev. Learn. Org. Int. J..

[2] Saleh A, Watson R (2017). Business excellence in a volatile, uncertain, complex and ambiguous environment (BEVUCA). TQM J..

[3] Kaivo-oja JRL, Lauraeus IT (2018). The VUCA approach as a solution concept to corporate foresight challenges and global technological disruption. Foresight.

[4] Spady WG (1988). Organizing for results: The basis of authentic restructuring and reform. Educ. Leadersh..

[5] Kaliannan DM, Chandran SD (2012). Empowering students through outcome-based education (OBE). Res. Educ..

[6] Syeed MM, Shihavuddin A, Uddin MF, Hasan M, Khan RH (2022). Outcome based education (OBE): Defining the process and practice for engineering education. IEEE Access.

[7] Zamir MZ, Abid MI, Fazal MR, Qazi MAAR, Kamran M (2022). Switching to outcome-based education (OBE) system, a paradigm shift in engineering education. IEEE Trans. Educ..

[8] Tan K, Chong MC, Subramaniam P, Wong LP (2018). The effectiveness of outcome based education on the competencies of nursing students: A systematic review. Nurse Educ. Today.

[9] Li C, Wang B (2015). Impact of student extracurricular activity involvement on student leadership: Mediating effect of general self-concept. J. Shanghai Univ. Sport.

[10] Bandura A (2001). Social cognitive theory: An agentic perspective. Annu. Rev. Psychol..

[11] Kautish P, Hameed S, Kour P, Walia S (2022). Career beliefs, self-efficacy and VUCA skills: A study among generation Z female students of tourism and hospitality. J. Hosp. Leis. Sport Tour. Educ..

[12] Killen, R. *Outcomes-Based Education: Principles and Possibilities.* University of Newcastle. https://academic.payap.ac.th/pyu/uploads/userfiles/file/KM/2558/1_-2-Killen_paper_good-kena-baca1.pdf (2000). Accessed October 26, 2022.

[13] Datta R (2021). Development of a portfolio framework for implementation of an outcomes-based healthcare professional education curriculum using a modified e-Delphi method. Med. J. Armed Forces India.

[14] Slavcev RA, Tjendra J, Cheung D (2013). A model of iterative outcome-based curriculum design and assessment for strategic pharmacy education in Canada. Curr. Pharm. Teach. Learn..

[15] Jadhav MR, Kakade AB, Jagtap SR, Patil MS (2020). Impact assessment of outcome based approach in engineering education in India. Procedia Comput. Sci..

[16] Rathy GA, Sivasankar P, Gnanasambandhan TG (2020). Developing a knowledge structure using outcome based education in power electronics engineering. Procedia Comput. Sci..

[17] Baguio J (2019). Outcomes-based education: Teachers’ attitude and implementation. Univ. Bohol Multidiscip. Res..

[18] Arcodia C, Dickson C (2009). ITHAS: An experiential education case study in tourism education. J. Hosp. Tour. Educ..

[19] Felicen SS (2021). Effectiveness of the implementation of outcomes based education (OBE) in the college of international tourism and hospitality management. Asia Pac. J. Educ..

[20] Berlach RG, O'Neill M (2008). Western Australia's English course of study: To OBE or not to OBE, perhaps that is the question. Aust. J. Educ..

[21] Shaheen S (2019). Theoretical perspectives and current challenges of OBE framework. Int. J. Eng. Educ..

[22] Kinsinger, P. & Walch, K. *Living and Leading in a VUCA World*. Thunderbird School of Global management, Arizona State University, Phoenix, Arizona. http://knowledgenetwork.thunderbird.edu/research/2012/07/09/kinsinger-walch-vuca/ (2012). Accessed November 17, 2022.

[23] Lawrence K. *Developing Leaders in a VUCA Environment*. UNC Executive Development. http://www.growbold.com/2013/developing-leaders-in-a-vucaenvironment_UNC.13.pdf.(2013). Accessed November 17.

[24] Laukkonen RE, Biddel H, Gallagher R (2019). Preparing Humanity for Change and Artificial Intelligence: Learning to Learn as a Safeguard Against Volatility, Uncertainty, Complexity, and Ambiguity.

[25] Hadar LL, Ergas O, Alpert B, Ariav T (2020). Rethinking teacher education in a VUCA world: Student teachers’ social-emotional competencies during the COVID-19 crisis. Eur. J. Teach. Educ..

[26] Intagliata J, Small D (2005). McDonald’s Corporation: A Customized Leadership Development Program Targeted to Prepare Future Regional Managers.

[27] Horstmeyer A (2020). The generative role of curiosity in soft skills development for contemporary VUCA environments. J. Org. Change Manag..

[28] Shavelson RJ, Hubner JJ, Stanton GC (1976). Self-concept: Validation of construct interpretations. Rev. Educ. Res..

[29] Turner JC, Hogg MA, Oakes PJ, Reicher SD, Wetherell MS (1987). Rediscovering the social group: A self-categorization theory. Basil Blackwell.

[30] Arens AK, Yeung AS, Craven RG, Hasselhorn M (2011). The twofold multi-dimensionality of academic self-concept: Domain specificity and separation between competence and affect components. J. Educ. Psychol..

[31] Kadir MS, Yeung AS, Diallo TM (2017). Simultaneous testing of four decades of academic self-concept models. Contemp. Educ. Psychol..

[32] Wren JT (1994). Teaching leadership: The art of the possible. J. Leadersh. Stud..

[33] Spady, W. G. *Outcome-Based Education: Critical Issues and Answers* (American Association of School Administrators, 1994). https://files.eric.ed.gov/fulltext/ED380910.pdf. Accessed October 26, 2022.

[34] Valizadeh S, Mohammadpour Y, Parvan K, Lakdizaji S (2009). The effect of outcome-based education on nursing students’ clinical competency. Iran. J. Med. Educ..

[35] Yildiz K (2022). Experiential learning from the perspective of outdoor education leaders. J. Hosp. Leis. Sport Tour. Educ..

[36] Chiou CC, Tien LC, Tang YC (2020). Applying structured computer-assisted collaborative concept mapping to flipped classroom for hospitality accounting. J. Hosp. Leis. Sports Tour. Educ..

[37] Lu HF (2021). Enhancing university student employability through practical experiential learning in the sport industry: An industry-academia cooperation case from Taiwan. J. Hosp. Leis. Sport Tour. Educ..

[38] Stefanou C, Stolk JD, Prince M, Chen JC, Lord SM (2013). Self-regulation and autonomy in problem and project-based learning environments. Act. Learn. High. Educ..

[39] Stevens NG, Peltier GL (1994). A review of research on small-school student participation in extracurricular activities. J. Res. Rural Educ..

[40] Adeyemo SA (2010). The relationship between students participation in school based extracurricular activities and their achievement in physics. Int. J. Sci. Technol. Educ. Res..

[41] Trautwein U, Möller J, Lipnevich AA, Preckel F, Roberts RD (2016). Self-concept: Determinants and consequences of academic self-concept in school contexts. Psychosocial Skills and School Systems in the 21st Century: Theory, Research, and Practice.

[42] Blomfield CJ, Barber BL (2011). Developmental experiences during extracurricular activities and Australian adolescents’ self-concept: Particularly important for youth from disadvantaged schools. J. Youth Adolesc..

[43] Arcodia C, Novais MA, Cavlek N, Humpe A (2020). Educational tourism and experiential learning: Students’ perceptions of field trips. Tour. Rev..

[44] Zhai X, Gu J, Liu H, Liang JC, Tsai CC (2017). An experiential learning perspective on students’ satisfaction model in a flipped classroom context. J. Educ. Technol. Soc..

[45] Wu HC, Cheng CC, Ai CH (2018). A study of experiential quality, experiential value, trust, corporate reputation, experiential satisfaction and behavioral intentions for cruise tourists: The case of Hong Kong. Tour. Manag..

[46] Stiehm, J. H. *The U.S. Army War College: Military Education in a Democracy*. https://digital-commons.usnwc.edu/cgi/viewcontent.cgi?article=2305&context=nwc-review (2002). Accessed October 26, 2022.

[47] Boe O, Säfvenbom R, Johansen RB, Buch R (2018). The relationships between self-concept, self-efficacy, and military skills and abilities. Int. J. Learn. Teach. Educ. Res..

[48] Marsh HW, Pekrun R, Murayama K, Arens AK, Parker PD, Guo J, Dicke T (2018). An integrated model of academic self-concept development: Academic self-concept, grades, test scores, and tracking over 6 years. Dev. Psychol..

[49] Choy MW, Yeung AS (2022). Cognitive and affective academic self-concepts: Which predicts vocational education students’ career choice?. Int. J. Educ. Res. Open..

[50] Atitsogbe KA, Moumoula IA, Rochat S, Antonietti JP, Rossier J (2018). Vocational interests and career indecision in Switzerland and Burkina Faso: Cross-cultural similarities and differences. J. Vocat. Behav..

[51] Grigg S, Perera HN, McIlveen P, Svetleff Z (2018). Relations among math self efficacy, interest, intentions, and achievement: A social cognitive perspective. Contemp. Educ. Psychol..

[52] Olivier E, Archambault I, De Clercq M, Galand B (2019). Student self-efficacy, classroom engagement, and academic achievement: Comparing three theoretical frameworks. J. Youth Adolesc..

[53] Arens AK, Bodkin-Andrews G, Craven RG, Yeung AS (2014). Self-concept of Indigenous and non-Indigenous Australian students: Competence and affect components and relations to achievement. Learn. Individ. Differ..

[54] Arens AK, Marsh HW, Craven RG, Yeung AS, Randhawa E, Hasselhorn M (2016). Math self-concept in preschool children: Structure, achievement relations, and generalizability across gender. Early Child. Res. Q..

[55] Yeung AS, Craven RG, Ali I (2013). Self-concepts and educational outcomes of Indigenous Australian students in urban and rural school settings. Sch. Psychol. Int..

[56] Jansen M, Lüdtke O, Schroeders U (2016). Evidence for a positive relation between interest and achievement: Examining between-person and within-person variation in five domains. Contemp. Educ. Psychol..

[57] Markus H, Nurius P (1986). Possible selves. Am. Psychol..

[58] Bagozzi RP, Yi Y (1988). On the evaluation of structural equation models. J. Acad. Mark. Sci..

[59] Fornell C, Larcker DF (1981). Structural equation models with unobservable variables and measurement error: Algebra and statistics. J. Mark. Res..

